# The Implementation of a Rib Fracture Pathway at a Small District General Hospital to Improve Patient Care

**DOI:** 10.7759/cureus.38863

**Published:** 2023-05-11

**Authors:** Justin Collis, Barnaby Farquharson, Shirley Chan, Richard Dickson-Lowe

**Affiliations:** 1 General Surgery, Medway Maritime NHS Foundation Trust, Gillingham, GBR

**Keywords:** acute pain, stumbl, anaesthetic, general trauma surgery, rib fractures

## Abstract

Background and objective

Rib fractures are common presentations to the emergency department following blunt thoracic trauma. Despite this injury causing significant morbidity and mortality, no national guidelines exist to guide the acute management of this condition. In light of this, this quality improvement project was conducted at a district general hospital (DGH) with the aim of assessing the impact of using a simple rib fracture management pathway.

Methods

A retrospective review of paper notes and electronic databases of patients with a recorded diagnosis of "rib fractures" were reviewed. Following this, a management pathway was designed and then implemented, which incorporated BMJ Best Practices and local hospital needs. The study then assessed the impact of the pathway.

Results

Prior to implementing the pathway, a total of 47 individual patients were included in the statistical analysis. Of the patients analysed, 44% were older than 65 years. Of note, 89% received regular paracetamol for analgesia, 41% received regular nonsteroidal anti-inflammatory drugs (NSAIDs), and 69% received regular opioids. Advanced analgesics such as patient-controlled analgesia (PCA) and nerve blocks were poorly used; for instance, a PCA was used in only 13% of cases. Only 6% of patients received daily pain team reviews and only 44% of patients were seen by physiotherapists within the first 24 hours. Additionally, 93% of patients who were admitted under general surgery had a STUMBL (STUdy of the Management of BLunt chest wall trauma) prognostic score >10.

Post-pathway implementation, a total of 22 individual patients were included in the statistical analysis. Of them, 52% were older than 65 years. The use of simple analgesia was unchanged. However advanced analgesia was better escalated, and PCAs were used 43% of the time. The involvement of other healthcare professionals improved; 59% were reviewed by the pain team in the first 24 hours, 45% received daily pain team reviews, and 54% received advanced analgesia.

Conclusion

Based on our findings, implementing a simple rib fracture pathway is effective at improving the management of rib fracture patients admitted to our DGH.

## Introduction

Thoracic injury following trauma is a common presentation to accident and emergency (A&E) globally [1]. Concomitant rib fractures are estimated to occur in 20% of all thoracic trauma cases [2]. Rib fractures are often associated with acute damage to the underlying internal organs and structures such as haemothorax and pneumothorax, and may cause cardiac contusions. Data published by the Trauma Audit Research Network (TARN) in 2017 demonstrated that the thorax is the second most injured anatomical region in patients older than 60 years [3]. Despite increasing evidence that rib fractures have significant implications for morbidity and mortality, particularly in the elderly population [1,4,5], no national guidelines exist for their management.

A common sequela of rib fractures is pain, which can lead to pulmonary complications due to restricted ventilation. Pain impairs the ability to cough and take deep breaths, which leads to the accumulation of lung secretions, thereby predisposing individuals to basal atelectasis and pneumonia [5]. Early effective analgesia can preserve pulmonary function and avoid potentially life-threatening complications associated with a restricted tidal volume, such as pneumonia or invasive ventilation.

A fundamental component of effective rib fracture management is a multidisciplinary team (MDT) approach. The involvement of other specialties such as physiotherapy, pain team, geriatricians, surgeons, and dieticians lead to better outcomes [6]. As a result, the parent specialty should be able to easily identify how to engage these different specialties.

The primary aim of this quality improvement project was to assess whether the implementation of a simple rib fracture management pathway (Appendix 1) could improve adherence to best practices.

## Materials and methods

We conducted a retrospective cohort analysis, identifying patients with the diagnosis of rib fracture over a one-year period from May 2019 to May 2020. All patients referred to the general surgery team with a confirmed diagnosis of rib fractures either on X-ray or CT were included in the analysis. Data from patients with paper notes with missing or incomplete drug charts were excluded.

Paper notes and electronic databases of patients with a recorded diagnosis of "rib fractures" were reviewed. Paper notes were requested from the health records team within the audit department. We collected data points around baseline characteristics and the mechanism of injury. Our primary data points were based on BMJ Best Practice guidance [7], analgesia strategy, pain team review, dietician review, whether a nerve block was used, and physiotherapy involvement.

Additionally, our project used the STUMBL (STUdy of the Management of BLunt chest wall trauma) [8] score (Appendix 1) to determine prognosis, and such data points to calculate this score were collected, including the number of rib fractures, age, pre-injury anticoagulation, chronic lung disease, and recorded oxygen saturation on room air. Although there are many prognostic scoring systems available, we chose the STUMBL score because it has the highest sensitivity and specificity for predicting outcomes following rib fractures.

Following the initial cycle of data collection, a draft pathway was designed. The results and the pathway were then presented at multiple different departmental meetings, general surgery audit meetings, the trust monthly trauma meeting, and the trust management medicine committee group. Additionally, copies of the pathway were emailed to the physiotherapy lead, the pain team, and the frailty team. Feedback generated from these avenues of communication was acted upon and appropriate changes were made to the pathway. The pathway was then uploaded to the trust intranet where it could be accessed by all clinicians.

We then performed a second cycle of data collection over a shorter time period: from October 2021 to April 2022. The same data points and inclusion/exclusion criteria were used for the second cycle. An Excel proforma was created to streamline data collection. The Excel proforma was then merged and entered into the IBM SPSS Statistics analysis package (IBM Corp., Armonk, NY). Chi-squared testing was used to test for statistical differences.

## Results

During the first cycle, a total of 47 individual patient notes were reviewed retrospectively; they had been admitted under the general surgical department over a one-year period (May 2019-May 2020). In the second cycle, over a seven-month period (Oct 21-April 22), 22 individual patients were included in the analysis. The most common mechanism of injury was a fall from height (n=19, 40%) in the first cycle, whereas in the second cycle, the most common mechanism was road traffic accidents (n=11, 50%). In the initial cycle, 26 (56%) patients were younger than 65 years, 15 (31%) were older than 65 years, and six (13%) were older than 85 years. Similarly, in the second cycle, 12 (55%) were younger than 65 years, six (27%) were older than 65 years, and four (18%) were older than 85 years. The average length of admission in both cycles was five days.

**Figure 1:**
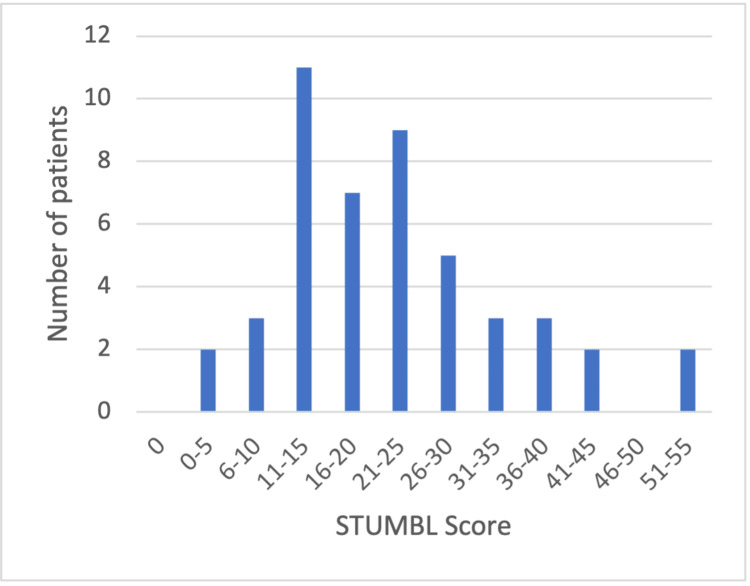
Histogram of STUMBL scores from cycle 1 STUMBL: STUdy of the Management of BLunt chest wall trauma

**Figure 2:**
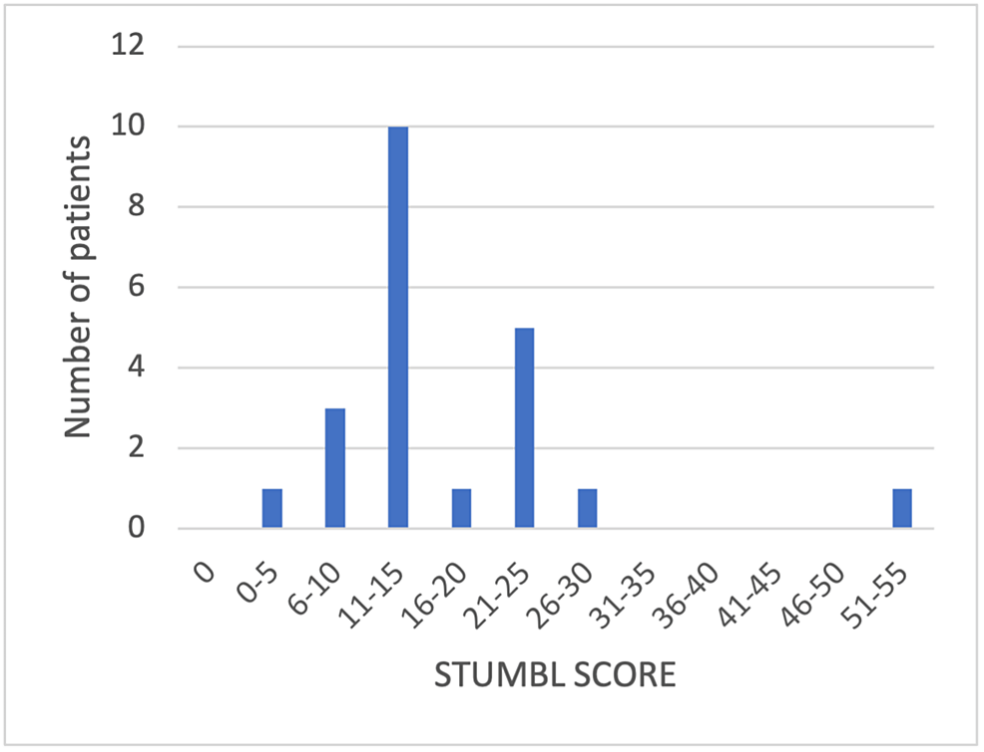
Histogram of STUMBL scores from cycle 2 STUMBL: STUdy of the Management of BLunt chest wall trauma

A high proportion of patients in both cycles had a high STUMBL score. In cycle 1, 42 (89%) patients had a score >10, and 17 (36%) had a score >25. In cycle 2, 18 (81%) patients had a score >10, and two (9%) had a score greater than 25. The distribution of scores is represented by the histograms presented in Figure 1 and Figure 2.

Analgesia forms an important component of rib fracture management. Analgesia strategies follow the WHO Analgesic Ladder. Our results demonstrated that as an organization we are good at starting low on the WHO analgesia ladder. In cycle 1, paracetamol was prescribed for 41 (87%) patients, nonsteroidal anti-inflammatory drugs (NSAIDs) for 19 (41%) patients, and regular opiates for 32 (69%) patients. In cycle 2, paracetamol was prescribed for 20 (91%) patients, NSAIDs for 12 (56%) patients, and regular opiates for 18 (82%) patients. 

However, in cycle 1, escalation of analgesia was poorly performed; patient-controlled analgesia (PCA) was used in six (13%) of patients, regional catheter block in two (4%), and epidural in three (6%) patients. This improved significantly in cycle 2: PCAs were used in nine (43%) patients, regional catheter blocks in seven (30%), and epidural blocks in two (8%) patients.

Engagement of the multidisciplinary team (MDT) was notably poorly executed in cycle 1; physiotherapy reviewed 21 (45%) patients within the first 24 hours, the pain team only reviewed three (6%) patients daily, and advanced analgesia requiring anesthetic support was only used in seven (15%) cases. In cycle 2, physiotherapy review within the first 24 hours was provided to 59% of cases (p=0.146), daily pain team review increased to 45% of cases (p=0.002), and advanced analgesia requiring anesthetic support improved to 54% of cases (p=0.003). A comparison of this is demonstrated in Table 1.

**Table 1:** Comparison of MDT involvement between the two cycles MDT: multidisciplinary team

		Total number of patients in cycle 1: 42				Total number of patients in cycle 2: 22		
	Number of patients in cycle 1 with the below treatment		%		Number of patients in cycle 2 with the below treatment		%	
Physiotherapy	21		44.7%		13		59.1%	
Pain team	3		6.4%		10		45.5%	
Advanced analgesia	7		14.9%		12		54.5%	

## Discussion

The aim of our quality improvement project was to assess and improve the management of rib fractures at our district general hospital (DGH). The initial cycle identified key areas for improvement, and we adapted our pathway design based on these needs.

Battle et al. developed and validated the STUMBL prognostic score for rib fractures. Their study found that a STUMBL score of 11 or greater was associated with a 30% chance of developing complications [1]. Our study found that greater than 80% of patients presenting had a STUMBL prognostic score of greater than 10. This demonstrates that clinicians are identifying and admitting high-risk patient cohorts. Additionally, it demonstrates that many patients admitted to our institution are at risk of developing complications requiring good supportive care.

A key finding related to our demographics was that in both cycles, patients aged 65 years and older represented greater than 40% of individuals who sustain rib fractures. This is an important statistic to be aware of because research demonstrates that complications associated with rib fractures, such as pneumonia and admission to intensive care, increase with age [9,10]. Bulgar et al. demonstrated in their research that patients above the age of 65 years were associated with two-fold mortality and morbidity when admitted for rib fractures [11]. Our project recognised this and we were able to discuss the results with the geriatric team. This allowed the pathway to incorporate a means of contacting the acute frailty team to provide input on this patient cohort, thereby ensuring that the care of these patients is optimised.

A fundamental component of rib fracture care is effective multimodal analgesia [12]. Patients are susceptible to pneumonia typically due to restricted tidal volume, which usually occurs 48-72 hours after injury [13]. This project found that the escalation of analgesia was performed poorly in the initial cycle, which is a common phenomenon within the medical profession [14,15,16]. Our strategy to improve this was to provide a template of suggested drugs to prescribe, improve access to the pain team, and signpost how to get nerve blocks/regional anesthesia (Appendix 1). Evidence indicates that nerve blocks are superior to systemic opioids in lowering the incidence of pulmonary complications in the context of rib fractures [17]. Although our strategy significantly improved the escalation of analgesia, there is still room for improvement in utilising blocks. Organising blocks at our intuition is challenging as it is often facilitated by the CEPOD anesthetist who may be busy in the theatre or unable to provide a block. A recommendation this study makes is the introduction of a "block fellow".

Physiotherapy forms a cornerstone of rib fracture management. Respiratory physiotherapy preserves pulmonary function through techniques such as assisted cough and incentivised spirometry [14,15]. In the first cycle, the physiotherapy team saw only 42% of patients. To address this, the pathway incorporated physiotherapy as an important part of the management and provided the contact details for the physiotherapy team. This action increased review in the first 24 hours to 59% of patients. Discussions with the physiotherapy team revealed that they were often unaware of these patients in the ward. A way they suggested to improve this was to include the diagnosis of rib fractures in the nurse handover sheet, which is where they get their initial clinical information from.

Rib fractures account for a large cohort of cases that are admitted under the general surgical team for management in DGHs. Furthermore, chest injuries are associated with 25% of deaths after trauma and have significant implications for morbidity, with survivors often losing working days as well as experiencing impaired functional capacity [16,17]. Therefore, early efficacious management of this injury provides an opportunity to mitigate these significant consequences.

A limitation of this project was the lower number of cases included in the second cycle, which predisposes the results to an element of bias. However, the number in the second cycle provides an accurate sample of the broad trend. Conducting a repeat cycle would be beneficial to ensure that the trend in improvement continues.

## Conclusions

The principle of managing rib fractures relies on the early identification of patients at risk of complications, multimodal pain management, and MDT engagement. Although there is a high prevalence of this injury, no national guidelines exist to help with the management strategy. 

Rib fractures are a significant cause of morbidity and mortality in the UK. A common sequela of rib fractures is pneumonia caused by impaired ventilation secondary to pain. The first cycle of the audit identified that analgesia was often escalated poorly, despite a high proportion of patients being at risk of complications based on their STUMBL scores. Furthermore, members of the MDT required to preserve pulmonary function did not review these patients on a regular basis. The project addressed these shortcomings through the design and implementation of a rib fracture pathway. Following the use of this pathway, there was a notable improvement in analgesia escalation and MDT engagement. This study demonstrates that simple care pathways can improve physician compliance with best practices and ultimately improve patient care.

## Figures and Tables

**Figure 3:**
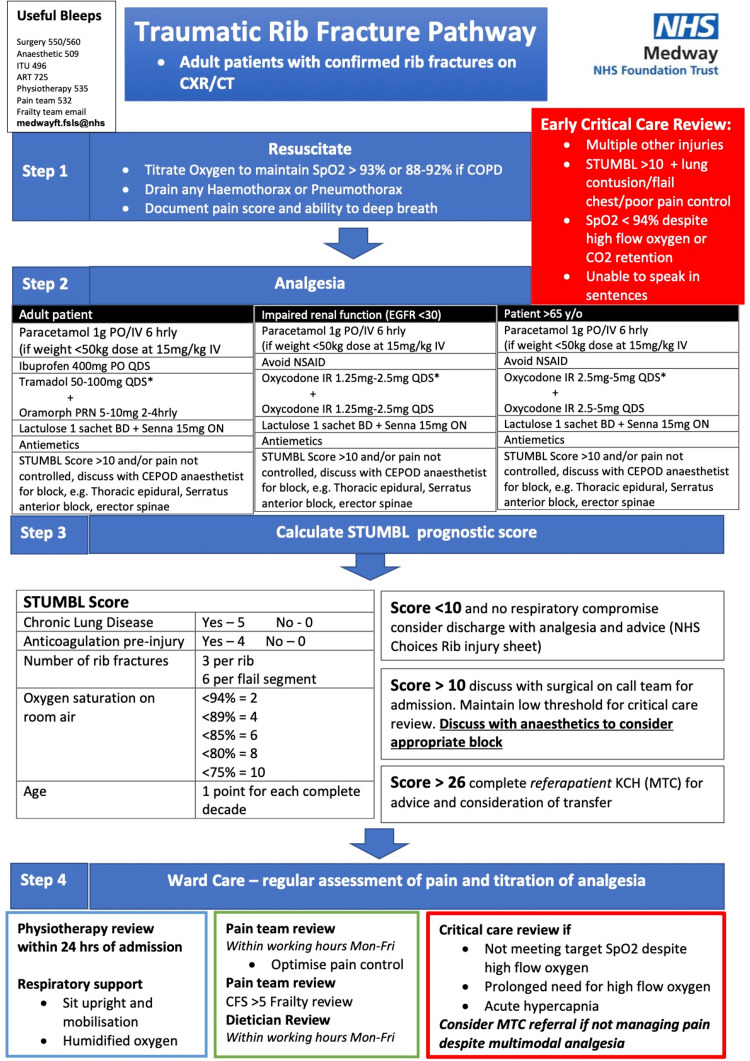
Traumatic rib fracture pathway
